# Wearable Sensor System to Monitor Physical Activity and the Physiological Effects of Heat Exposure

**DOI:** 10.3390/s20030855

**Published:** 2020-02-06

**Authors:** Sean Pham, Danny Yeap, Gisela Escalera, Rupa Basu, Xiangmei Wu, Nicholas J. Kenyon, Irva Hertz-Picciotto, Michelle J. Ko, Cristina E. Davis

**Affiliations:** 1 Department of Mechanical and Aerospace Engineering, University of California, Davis, CA 95616, USA; sapham@ucdavis.edu (S.P.); dyeap@ucdavis.edu (D.Y.); 2 Center for Healthcare Policy and Research, University of California, Davis, CA 95616, USA; gescalera@ucdavis.edu; 3 California Environmental Protection Agency, California Office of Environmental Health Hazard Assessment, 1515 Clay Street, Oakland, CA 94612, USA; Rupa.Basu@oehha.ca.gov (R.B.); Xiangmei.Wu@oehha.ca.gov (X.W.); 4 Department of Internal Medicine, 4150 V Street, Suite 3400, University of California, Davis, Sacramento, CA 95817, USA; njkenyon@ucdavis.edu; 5 Center for Comparative Respiratory Biology and Medicine, University of California, Davis, CA 95616, USA; 6 VA Northern California Health Care System, 10535 Hospital Way, Mather, CA 95655, USA; 7 Division of Environmental and Occupational Health, Department of Public Health, University of California, Davis, CA 95616, USA; iher@ucdavis.edu; 8 Center for Regional Change, Department of Public Health, University of California, Davis, CA 95616, USA; mijko@ucdavis.edu

**Keywords:** wearable physiological sensors, heart rate, skin temperature, activity monitoring, galvanometric response, personalized medicine, telehealth

## Abstract

Mobile health monitoring via non-invasive wearable sensors is poised to advance telehealth for older adults and other vulnerable populations. Extreme heat and other environmental conditions raise serious health challenges that warrant monitoring of real-time physiological data as people go about their normal activities. Mobile systems could be beneficial for many communities, including elite athletes, military special forces, and at-home geriatric monitoring. While some commercial monitors exist, they are bulky, require reconfiguration, and do not fit seamlessly as a simple wearable device. We designed, prototyped and tested an integrated sensor platform that records heart rate, oxygen saturation, physical activity levels, skin temperature, and galvanic skin response. The device uses a small microcontroller to integrate the measurements and store data directly on the device for up to 48+ h. continuously. The device was compared to clinical standards for calibration and performance benchmarking. We found that our system compared favorably with clinical measures, such as fingertip pulse oximetry and infrared thermometry, with high accuracy and correlation. Our novel platform would facilitate an individualized approach to care, particularly those whose access to healthcare facilities is limited. The platform also can be used as a research tool to study physiological responses to a variety of environmental conditions, such as extreme heat, and can be customized to incorporate new sensors to explore other lines of inquiry.

## 1. Introduction

Heat-related diseases are significant causes of mortality and morbidity for various populations, including civilian and military groups, and pose serious public health concerns. Increased ambient temperatures increase risks of not only heat illness and dehydration, but also ischemic heart disease, arrhythmias, renal failure, pneumonia and intestinal infections [1]. With anticipated global temperature shifts and growth of urban landscapes, heat-related injuries are likely to increase [2]. Though marginalized populations have a particular vulnerability to heat-related mortality, heat risk is widespread, affecting workplace health as well as military and athletic training. 

Athletes contribute a significant fraction of heat-related morbidity, especially amongst US high school athletes [3]. For athletes, exertional heat illness (EHI) is the most prominent cause of mortality [4]. EHI is preventable, but prevention is dependent on recognition of key symptoms and physiological changes. Without intervention, EHI can develop into severe and life-threatening exertional heat stroke [5]. Hydration is key to avoiding incidents of EHI, but even in well-hydrated athletes, extreme heat exposure has significant physiological effects [6]. Acute heat exposure is detrimental to muscle endurance, leaving athletes who train in hot weather especially susceptible to EHI [6]. Heat acclimation is recommended for athletes who train in extreme temperatures. Adaptability to heat stress can be enhanced using short-term acclimation via controlled hyperthermia and dehydration in highly trained athletes under careful monitoring [7]. 

Similarly, EHI is significant among military personnel, who frequently train and perform operations in extreme climate conditions. While acute heat exposure contributes to EHI, continuous and passive heat effects over a prolonged period can adversely affect military troops [8,9]. The military takes measures to predict and protect its trainees from EHI using hourly heat indices. Updated indices, including wet-bulb dry temperature (WBDT) and relative humidity dry temperature (RHDT), inform commanders to implement heat illness prevention guidelines and reduce physical training activities to mitigate the onset of EHI [9]. However, even with these techniques for EHI prediction, the risk of heat-related illnesses still exists. The military still requires robust improvements for monitoring heat effects and tracking the occurrence of EHI [9,10].

Heat exposure in the workplace presents a serious threat to employee health and productivity, yet it is often overlooked [11]. The risk of heat illness can be exacerbated by workplace requirements, such as clothing, environment, and behavior [12]. Although exposed workers can benefit from acclimation to thermal settings, they cannot adapt well to intermittent and sudden heat events. To mitigate the risk of occupational heat-related illness, surveillance is necessary to assess and address workplace hazards [11]. Assessment of workplace conditions and their related physiological effects for construction workers was done using a suite of wearable sensors, and though study results provide insight into potentially unsafe conditions, more research is required to inform legislation on occupational reform [11,13].

The construction and agriculture industries introduce the greatest risk for heat-related fatality, and while heat studies regarding agricultural workers are extensive, heat exposure research for construction is lacking [14]. Though heat illness may be the immediate concern to occupational health, heat strain and volume depletion that agricultural workers experience during shifts can result in acute kidney injury (AKI) [15]. The risk of heat-related AKI manifestation was found to be associated with increased strain based on the strenuous nature of the work [15]. Because different farm tasks may require different levels of effort, risk varies for individual workers. To characterize individual AKI risk, personalized health assessment may be necessary. A personalized approach to occupational heat illness prevention and treatment also may incorporate assessments of metabolic and behavioral responses that affect employer and worker decisions [16].

Finally, elderly individuals are especially susceptible to heat-related illness, especially the most elderly, whose ability to moderate personal temperature is impaired [17]; these individuals are more likely to be in nursing homes or medical care facilities. Extreme heat events cause severe cardiovascular and respiratory complications in the elderly that, if untreated, may result in mortality [18]. Symptoms of heat illness typically go unnoticed in aging adults, who often report that they do not feel the effects of a heat wave [18]. Although it is well documented that elderly individuals experience greater risk of heat-related mortality, the individual factors that characterize personal heat exposure are still not fully detailed [19]. 

Wearable sensors have been used extensively in research to monitor physiological effects, but studies typically use a variety of monitoring systems or single-parameter devices. Existing wrist-type devices allow for convenient and continuous monitoring of heart rate in active laborers with minimal impedance [20]. However, single-parameter devices fail to benefit from a broader, holistic health overview. Multi-parameter systems typically requiring a suite of monitors may be cumbersome and obtrusive. There is a need for a fully integrated compact wearable system that provides comprehensive and flexible health monitoring. This manuscript shows a design and prototype of a cost-effective integrated armband for multi-parameter health monitoring tailored to tracking physiological changes—skin temperature, heart rate, blood oxygen saturation, galvanic skin response, and activity level—at rest, under heat stress, and/or during exercise. Ultimately, our wearable technology could be instrumental in establishing a “personalized medicine” platform for athletes, members of the military or aging adults and potentially contribute to worksite monitoring programs to safeguard the health of employees facing heat stress on the job.

## 2. Materials and Methods

**Figure 1:**
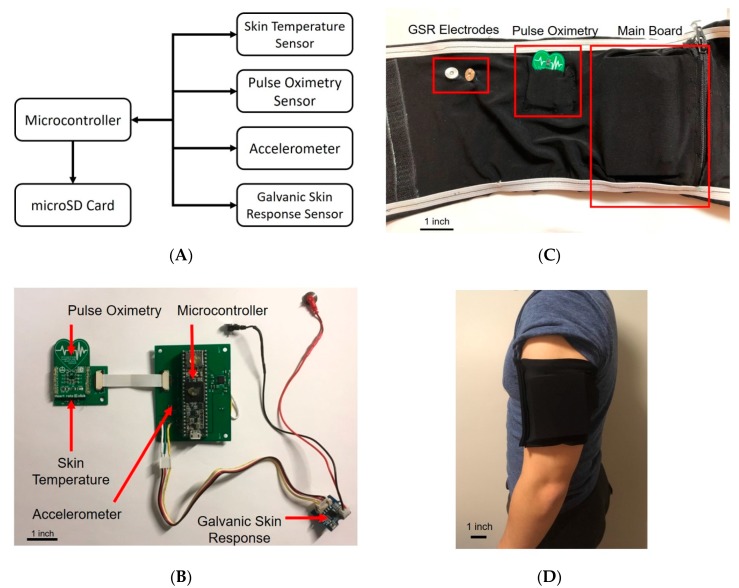
(**A**) The overall system diagram includes four commercial sensors integrated together using a commercial microcontroller; (**B**) The system components and PCBs are arranged prior to packaging; (**C**) The components are shown in the packaged armband; and (**D**) The final wearable device is shown.

To effectively monitor the physiological effects of heat, our wearable device includes sensors to facilitate measurement of heart rate, blood oxygen saturation, and skin temperature. Additionally, the device incorporates galvanic skin response, as a metric for skin moisture, and accelerometry for assessment of activity level. An integration diagram of the sensor suite is shown (Figure 1A). Briefly, these four different sensors were integrated into a single wearable format that is controlled by a simple commercial microcontroller.

### 2.1. Device Design and Development 

Two circuit boards (Figures S1 and S2) were designed around the Teensy 3.6 microcontroller module (Teensy 2016), which uses an ARM Cortex-M4F microprocessor from the NXP Semiconductors K66 family (NXP Semiconductors N.V., Eindhoven, Netherlands). The Teensy 3.6 also includes 1M Flash, 256K RAM, 4K EEPROM, as well as a built-in microSD card and mini USB ports. A 16 GB microSD card (SanDisk Corporation, Milpitas, CA, USA) was inserted into the Teensy for onboard data storage, and data were manually analyzed later off-line. 

Alongside the Teensy microcontroller, the main board includes the LIS2DH12 accelerometer (STMicroelectronics N.V., Amsterdam, Netherlands) and Grove GSR (Seeed Technology Co., Ltd., Shenzhen, China). The accelerometer registers motion along three axes with 2 g sensitivity and 10 Hz bandwidth to gauge activity intensity [21]. The internal analog to digital converter (ADC) outputs digital triaxial acceleration via I2C communication protocol. The commercial GSR sensor monitors changes in skin resistance due to changes in sympathetic response, namely changes in perspiration due to increased or decreased sympathetic activity. The voltage measured across two stainless steel disc electrodes (Cadwell Industries, Inc., Kennewick, WA, USA) is amplified using three LM324 Operational Amplifiers (Texas Instruments Inc., Dallas, TX, USA) on the commercial GSR module. The sensitivity of the device can be modulated using the built-in potentiometer, whose resistance can vary between 50–500 kΩ. The analog signal is read by the ADC on the Teensy 3.6 using an internal voltage reference of 3.0 V from an onboard voltage reference source. 

The peripheral board incorporates a MLX90614 medical accuracy infrared thermopile (Melexis N.V., Ypres, Belgium) and the Heart Rate 3 Click module (Mikroelektronika LLC, Belgrade, Serbia), which uses the AFE4404 (Texas Instruments Inc., Dallas, TX, USA) as the analog front end for the SFH7050 (OSRAM Licht AG, Munich, Germany) pulse oximetry sensor. The LED array of the pulse oximetry sensor can output three wavelengths: infrared at 950 nm, red at 660 nm, and green at 525 nm. Infrared and red LEDs are utilized to enable blood oxygen saturation calculation. The green LED was omitted for battery life management. Infrared thermometry was chosen for the device because of its performance and utility in other commercially available medical devices. 

The boards are powered by a 3.7 V, 2500 mAh Adafruit 328 lithium ion battery (Adafruit Industries, New York City, NY, USA) to achieve a minimum battery life of 48 h for the system and minimize wires/cables. Using 500 mA of charge current, the battery charges fully in 5 h. The power and data lines of the main board are connected to the peripheral board via a flat flexible cable, allowing the skin temperature and pulse oximetry sensors to interface directly with the skin to achieve accurate measurements. The main board along with the battery are housed in a phone-sized housing manufactured from Delrin^®^ (Dupont, Wilmington, DE, USA), and the entire package is fit into a compact armband the user can unobtrusively wear on the upper arm. 

Custom armband wear was intended to mimic commercial sports armbands for phones. This scheme allows for easy acclimation to the device, as well as improved core temperature estimation accuracy compared to wrist-type devices. A physical layout of the component parts before packaging is shown (Figure 1B); the packed armband is shown (Figure 1C); and the final wearable device is shown (Figure 1D). The total packaged size of the system is 3.356 inch × 2.965 inch × 1.05 inch, and the armband is 12.50 inch × 5.63 inch.

### 2.2. Human Subjects

This work was approved by the University of California, Davis human subjects review board under IRB 1396471-1. A total of 16 subjects between ages 21 to 54, including 10 males and 6 females, were fitted with our device and a commercial pulse oximeter. Participants were asked to perform 5 different activities in a single session while wearing the devices: sitting, climbing stairs, walking, jogging, and sprinting. Participants began by sitting for 20 min: the first 10 min allowed the temperature to equilibrate to the individual, and infrared temperature measurements were taken across the forehead concurrently, every minute for the last 10 min. Each participant then climbed stairs, walked, and jogged for 5 min each, before finishing with a 1 min sprint. 

### 2.3. Validation and Calibration Protocols

Once prototyped, our device was compared to standard clinical devices to calibrate and benchmark sensor performance. Skin temperature measurements were taken on the forehead using an iProvén DMT-489 infrared thermometer (iProvén, Rotterdam, The Netherlands), which served as surrogates for core body temperature. Heart rate and blood oxygen saturation were calculated from optical data using red-infrared photoplethysmography (PPG) and compared to measurements from a Contec CMS50D+ pulse oximeter (Contec Medical Systems Co., Ltd., Qinhuangdao, China). Data from these commercial monitors were taken concurrently with measurements from our device, so that models could be developed to relate device sensor data to data from their commercial counterparts. Calibration curves were generated to relate calculated device metrics to corresponding clinical measurements. This was performed across the full dynamic range of the sensors within the prototype.

The photodiode of the pulse oximetry sensor interfaces with a 22-bit ADC onboard the analog front end that has a full-scale input range of ±1.2 V. Feedback resistance was set to 10 kΩ to program the gain of the transimpedance amplifier; minimum amplifier gain was used to avoid augmentation of ambient noise. Drive current to red and infrared LEDs was tuned to 8 mA to ensure that the photodiode output avoids saturation of the ADC. Offset cancellation current from the digital-to-analog converter (DAC) was set to −7.0 μA to operate the ADC in a midscale range from 0.2–0.6 V. The system is operated at 50 Hz sampling frequency for consistency with commercial pulse oximetry. 

The temperature sensor has 16-bit resolution for 0.01 °C precision and a high accuracy dynamic range between 22–40 °C, making it suitable for physiological applications. The sensor capabilities allow it to capture minute variations in skin temperature, making it suitable for use in this device for monitoring temperature on the upper arm. Because skin temperature varies proportionally with core body temperature, the temperature sensor is used to estimate core body temperature. 

The accelerometer was configured for a sensitivity range between ±2 g with 1 mg resolution. These settings are consistent with typical human accelerometry applications, providing the ability to gauge activity intensity.

The 3.0 V reference and 10-bit resolution of the ADC correspond to a range of 1–16 μS with a resolution of 0.1 μS for the measurement of skin conductance. The capability of this commercial sensor was evaluated for its sensitivity to track changes in skin moisture as a result of perspiration. 

### 2.4. Data Analysis

**Figure 2:**
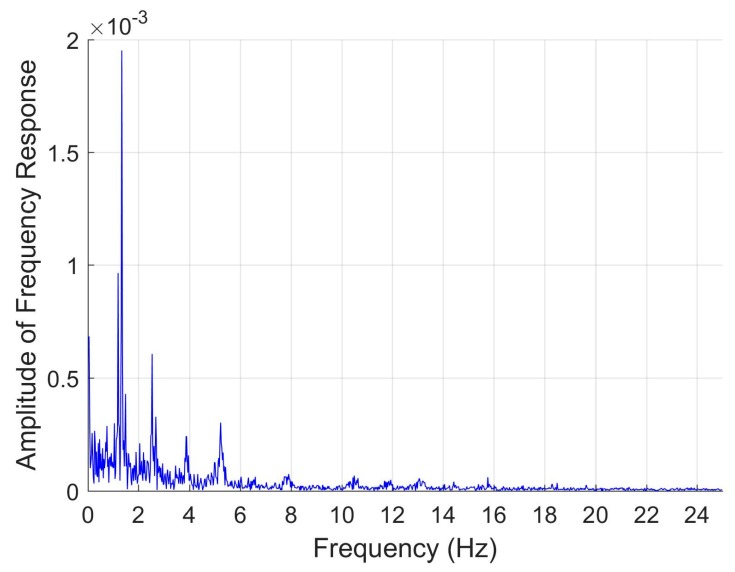
Single-sided amplitude spectrum from the Fast Fourier Transform of a single wavelength from a representative photoplethysmogram. Peak frequency occurs at a frequency of 1.342 Hz.

MatLab R2017 A (MathWorks, Inc., Natwick, MA, USA) software was used for processing, analyzing, and visualizing data. The optical waveforms from the pulse oximetry sensor were pre-processed using Fast Fourier Transform to obtain the single-sided spectrum frequency response. Fast Fourier Transform (FFT) is a common and robust technique for frequency analysis, especially using data with noise corruption. Applying FFT to the photoplethysmography (PPG) waveform converts the data to the frequency domain, which allows for maximum frequency components to be easily seen (Figure 2). Red and infrared PPG waveforms were used separately to calculate heart rate. Five minute datasets across a range of heart rates from 69–150 beats per minute (bpm) were subdivided into 40 s sections, and the MatLab FFT algorithm was used to convert the data subset to the frequency domain [22]. Our frequency range was bounded between 0.5–3.5 Hz to limit physiologically relevant heart rates to 30–210 bpm. Standard MatLab “max” functions were used to extract the peak frequency component within this range, which was used to calculate the heart rate. Heart rate was then averaged over 5 min for comparison to averaged heart rate measurements output from the reference commercial pulse oximeter, which were also averaged over a 5-min window.

(1) Heart Rate=frequency ×60

The photoplethysmogram consists of pulsatile alternating current (AC) and steady direct curent (DC) components. The ratio of AC to DC components for red and infrared wavelengths can be used together to calculate blood oxygen saturation [23]. The underlying DC level for each waveform was determined from the average of the data over 10 s intervals, and the AC component was averaged from the peak amplitudes for the same data subset.

(2) $$SpO_{2}=110-25\times R$$

(3)  R=ACredDCredACIRDCIR 

Standardized and device calculated saturation data were averaged separately and their standard deviations calculated for comparison. The average blood oxygen saturation from the commercial pulse oximeter was 97.5 ± 0.67; our device produced average blood oxygen saturation of 85.0 ± 0.010. Consistently lower blood oxygen saturation measurements suggested that our device measurements could be corrected using a bias term. We then sought to estimate bias by using the average difference between device and gold standard measurements across the entire data set. A Wilcoxon signed rank test was used to determine if corrected device values of blood oxygen saturation exhibited significant difference from gold standard measurements (*p* < 0.05). A similar biasing approach was used to estimate core body temperature from skin temperature measurements.

Linear regression was applied to develop models to relate device calculated heart rate to standardized pulse rate data. The correlation was calculated to assess the performance of this approach for both heart rate and temperature. A 95% confidence interval was determined for the calibration curve using the standard MatLab functions, and 95% prediction intervals were calculated to afford future interval estimates from individual observations:

(4) $${\hat{Y}}^{*}-t_{n-1,\;0.025}\;s\;\sqrt{1+\frac{1}{n}}\;\leq \;Y^{*}\leq \;{\hat{Y}}^{*}+t_{n-1,0.025}\;s\;\sqrt{1+\frac{1}{n}},$$

where *n* is the number of data points and *s* is the standard deviation of the original data set. This predicted value ± an error term is then reported for new sensor data.

For simple interpretation of accelerometry data, five levels of activity intensity were defined, and data classification was achieved by using support vector machine (SVM). SVM was chosen for its practicality with small datasets, robust performance with multi-feature classification, and utilization in activity recognition with accelerometry data. Multiclass SVM is a supervised machine learning algorithm that uses hyperplanes to divide a dataset. Training accelerometry data is labeled by intensity level for implementation of SVM. Maxima and minima for each axis are unique to each level of intensity, allowing them to serve as characteristic features for the accelerometry data. Each accelerometry dataset is defined by six features: minimum and maximum x-axis acceleration; minimum and maximum y-axis acceleration; minimum and maximum z-axis acceleration. To train and test the activity classification SVM, accelerometry datasets were used with a 70/30 split, where 70% of the data was used for training and 30% of the data was used for testing. Accelerometry datasets incorporated 30 s intervals of a representative activity, from which tri-axial minima and maxima were extracted using MatLab “min” and “max” functions. The labeled training set was fed into the MatLab multiclass SVM algorithm to define the five classes and divide data six-dimensionally. The model was then validated using the testing set to determine classification accuracy. Confusion matrices were generated from the test data to visualize classification error. 

## 3. Results

### 3.1. Heart Rate

**Figure 3:**
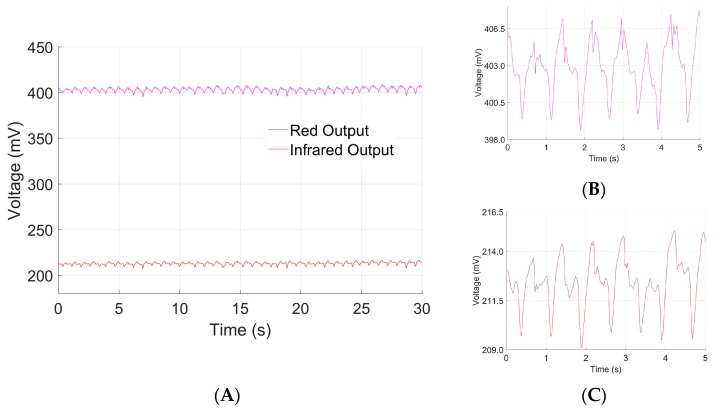
(**A**) The pulse oximetry sensor outputs a representative raw plethysmography waveform that shows the output from both infrared and red wavelengths. Magnified views of (**B**) infrared and (**C**) red are presented to show smaller scale features.

A red-infrared pulse oximetry scheme was utilized to monitor heart rate. The sensor from the armband outputs optical data from both red and infrared wavelengths in the form of a photoplethysmogram (Figure 3A). The typical PPG shape contains two main features: a primary systolic peak and a secondary diastolic peak [24]. The output PPG consists of waveforms from each red and infrared wavelengths (Figure 3B,C) that each exhibit features of a classical PPG waveform. In this armband scheme, pulse oximetry is administered on the upper arm, as opposed to usual sites on the finger or ear lobe. The vascular beds in the upper arm are deeper, so interference by skin and musculature may explain the deviations in PPG shape when compared to traditional finger pulse oximetry [25].

**Figure 4:**
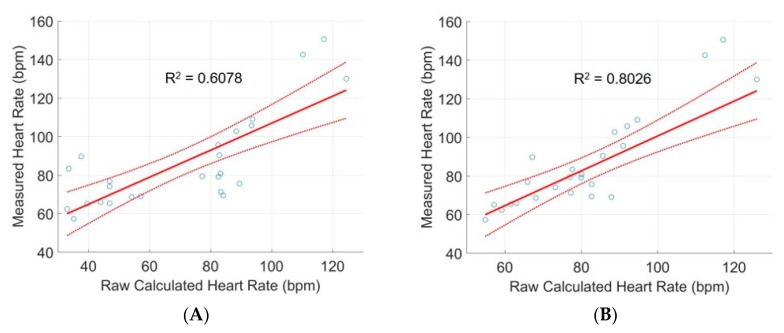
Linear regression models to compare the mean commercial sensor pulse oximetry rate to mean heart rate calculated using (**A**) red waveforms and (**B**) infrared waveforms from multiple test subjects. 95% confidence intervals are plotted for each regression model.

Although noise can be seen in the individual PPG waveforms (Figure 3B,C), pre-processing of the raw data using the MatLab FFT algorithm is capable of obtaining distinct frequency components; FFT must be applied to each PPG dataset to enable heart rate estimation. The heart rate calculated from PPG was compared to measurements provided by the commercial pulse oximeter taken at the same time. Linear regressions were produced for both red and infrared wavelengths to relate the calculated heart rate to the measured (Figure 4A,B). Heart rate calculated from infrared wavelength shows greater correlation (R^2^ = 0.8026) to the actual measurements than does red wavelength (R^2^ = 0.6078). The long wavelength of infrared light allows it to penetrate more deeply into the vessel beds of the upper arm; thus, it is expected that infrared wavelength estimates heart rate better [25]. Moreover, expected interference in the upper arm produces a relationship between device calculated heart rate and commercial measured heart rate that is not 1:1. However, the heart rate calculated from the infrared PPG waveform exhibits close correlation when modeled according to the linear regression, allowing for us to adjust our device’s measurement of heart rate to true heart rate. The linear fit for infrared wavelength to true heart rate is given by ${\hat{Y}}^{*}=1.2207x^{*}-12.3926$. The 95% prediction interval for a given observation is 97.5201 ± 23.9109, providing interval estimates for future device measurements.

### 3.2. Blood Oxygen Saturation 

Blood oxygen saturation was extracted from the AC and DC components of the red and infrared reflectance data of the PPG waveform [23] (Figure 3). The raw calculated blood oxygen saturation 85.0 ± 0.01 is consistently lower than the measured standard values 97.5 ± 0.67, suggesting that our device measurements of blood oxygen saturation are off by some bias term. To correct the raw values to better correspond to the measured, bias was calculated from the mean difference between measured and raw values. The average adjusted raw blood oxygen saturation, 97.5 ± 0.01, correlates with the average of measured values. A Student’s *t*-test demonstrated no significant difference between adjusted raw blood oxygen saturation and gold standard measurements of blood oxygen saturation (*p* < 0.05). The difference between raw calculated and measured blood oxygen saturation can be attributed to obstructed penetration of red wavelengths in the upper arm. Red light is susceptible to interference while infrared light is more resistant, contributing to the consistently lower measurements of blood oxygen saturation [25]. Variability of calculated blood oxygen saturation is narrower than that of measured values, indicating that the pulse oximetry sensor may be less capable of capturing small changes in blood oxygen saturation. For physiologically significant health indication, large changes in blood oxygen saturation are most significant; this scheme of armband pulse oximetry may serve as an approximation for blood oxygen saturation.

### 3.3. Skin Temperature 

**Figure 5:**
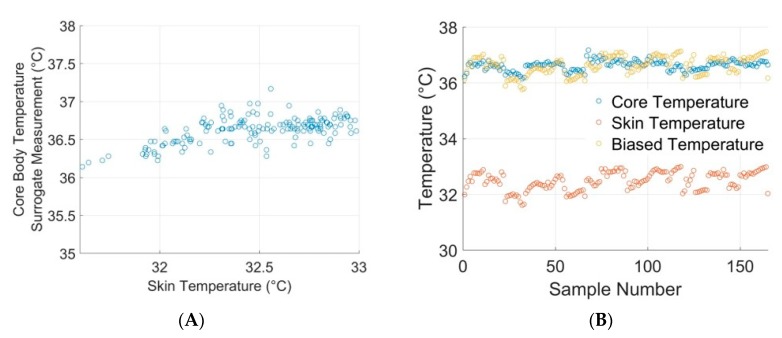
(**A**) Mean surrogate core body temperature is plotted against mean skin temperature from multiple subjects. (**B**) Raw skin temperature is biased, demonstrating good coincidence with surrogate core body temperature measurements.

Surrogate core temperature measurements are estimated using the infrared forehead thermometer taken to capture the physiological range and compared to corresponding skin temperature measurements on the upper arm from the device. The skin temperature is plotted against surrogate core body temperature to explore the relationship between the two (Figure 5A). Average skin temperatures of 32.48 ± 0.33 °C were reported for surrogate core body temperature measurements of 36.62 ± 0.17 °C. Because of the small variability of skin temperature in response to surrogate core body temperature, a bias term was determined from the mean difference between skin temperature and surrogate core body temperature. The calculated biased skin temperatures (36.62 ± 0.33 °C) generally show good agreement with surrogate core body temperature measurements (Figure 5B). These observations are corroborated by Wilcoxon signed rank test, which fails to demonstrate a statistically significant difference between biased skin temperature and surrogate core body temperature (*p* < 0.05). Additionally, small deviations in skin temperature are insignificant to overall health assessment; serious health indications, such as hypothermia and hyperthermia, are associated with extreme aberrations from normal body temperature. Thus, the ability of the device to track trends in estimated core body temperature and biasing of device skin temperature measurements provides a reasonable method for estimating core temperature.

### 3.4. Activity Classification

**Figure 6:**
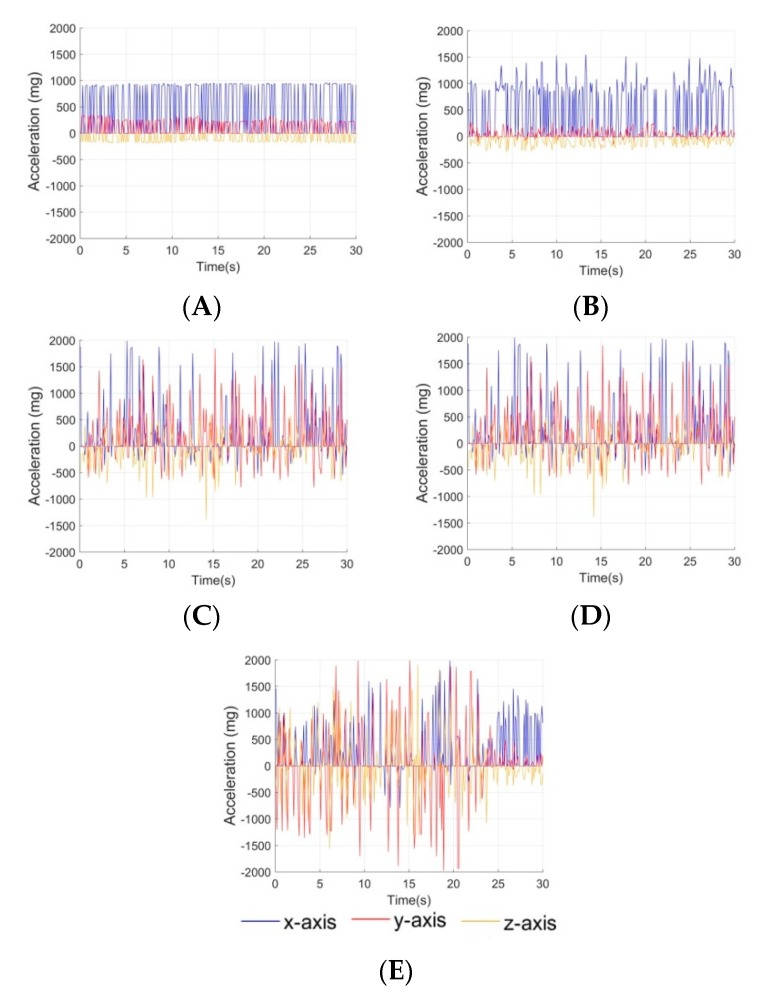
Representative triaxial accelerometry representing intensity levels: (**A**) level 1 sitting, (**B**) level 2 walking, (**C**) level 3 climbing stairs, (**D**) level 4 jogging, (**E**) level 5 sprinting.

Tri-axial accelerometry data was collected while subjects were performing activities representing different levels of intensity (Table 1). The x axis representing medial/lateral movement, the y axis representing anterior/posterior, and z representing superior/inferior vertical movement. In order to easily interpret the accelerometry data, a scheme to categorize activities based on intensity was investigated. Maxima and minima for each axis of accelerometry data were determined using intrinsic MatLab functions to generate unique features characteristic of each intensity level (Table 1). Six features were produced for each dataset: one minimum and one maximum for each x, y, and z axis. As intensity level increases (Figure 6A–E), the acceleration ranges for each axis also increase. The maxima and minima for each axis are distinct for each representative activity, making them useful as identifying features. These features and their associated intensities were used to train a support vector machine (SVM) MatLab algorithm for automated classification of data. Training and testing sets were compiled according to a 70/30 split of 50 accelerometry datasets. 

Once trained, the SVM classifier was validated using the testing set, producing an 87.5% success rate in labeling test data. Most false assignments occurred between levels 2 and 3, which demonstrate similar acceleration ranges; SVM struggles to define distinct hyperplanes between classes with similar features, so it is expected that most misclassification occurs between activities with similar accelerometery profiles. The performance of the SVM classifier can be visualized via a classification matrix (Table 2). Despite the classifier difficulty discerning between levels 2 and 3, it has success determining large differences in activity intensity, which is most significant to understanding dramatic changes in physiological effects. High accuracy of the model demonstrates that multiclass SVM serves as a reliable means for estimation of activity level. 

### 3.5. Galvanic Skin Response 

**Figure 7:**
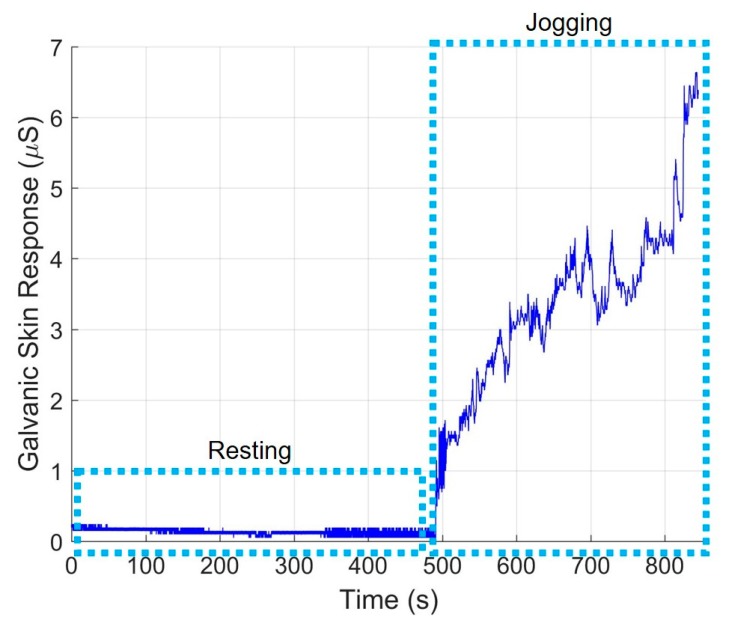
Representative galvanic skin response shows an increase in skin conductance as a result of perspiration during a jogging period.

Electrodes on the armband capture galvanic skin response on the upper arm. Measurements were taken during low and high activity periods to examine the sensitivity of the sensor to changes in perspiration. During exercise periods, the galvanic skin response sensor captures an increase in skin conductance as a result of increased perspiration (Figure 7). The device responds well to changes in skin conductance and can serve as a metric for relative skin hydration defined by variations from an individual’s baseline.

## 4. Discussion

Our new device design presents a proof-of-concept approach for an inexpensive custom alternative to bulky devices for wearable non-invasive, multi-parameter health monitoring. Using PPG, the device provides convenient estimations of heart rate and blood oxygen saturation. Though pulse oximetry is typically administered in regions with high vascular density and superficial vascular depth, the utility of pulse oximetry in an armband-type device affords convenience during exercise and demonstrates less sensitivity to motion artifacts [25]. Despite hindered accuracy from deep vessel beds, the device demonstrated ability to track trends in heart rate and good correlation to calibration via traditional finger-type pulse oximetry to afford us the capability of adjusting from device measurements of heart rate to true heart rate. Our scheme of pulse oximetry works better for low to moderate heart rates; linear regression analysis is less accurate for higher heart rates, likely due to increased activity and artifacts. Future schemes of the device may couple pulse oximetry with biopotential heart rate measurements via single or two lead ECG. This iteration of the device was designed for prolonged usage over a 48 h period, so biopotential sensors were avoided to minimize power consumption. Nevertheless, the utilization of armband pulse oximetry coupled with linear regression modeling provides clinicians with a gauge for instantaneous heart rate as well as changes in heart rate over time. Blood oxygen saturation can be also adequately estimated and adjusted to accurately reflect measurements from clinical pulse oximeters. 

Continuous monitoring via pulse oximetry is standard practice in critical care, and noninvasive measurements of arterial blood oxygen saturation are routine [26]. Commercial pulse oximeters utilize the combination of red and infrared light to penetrate into the cutaneous vascular bed [26]. The estimation of blood oxygen saturation is based on the difference in absorbance between oxygenated and deoxygenated hemoglobin–deoxygenated hemoglobin absorbs red light better whereas oxygenated hemoglobin absorbs infrared light better [26]. The ratio of the red to infrared absorbance, known as the modulation ratio, reflects the ratio of deoxygenated to oxygenated hemoglobin, which provides a metric for blood oxygen saturation [26]. The system is robust for fingertip or ear lobe applications, but is limited by light scattering, reflection, and absorbance by other components of blood and tissue [26]. Pulse oximetry is also susceptible to motion, so care must be taken to discern and filter out artifacts [27].

As mentioned, drastic changes to blood oxygen saturation represent serious health concerns. To ensure that the device can reflect low blood oxygen saturation, our device should ultimately be tested in hypoxic conditions, where hypoxemia would result in abnormally low blood oxygen. Traditional pulse oximetry is capable of good accuracy in tracking moderate to severe hypoxia, achieving blood oxygen measurements as low as 57% [28]. Ear lobe pulse oximetry performed better than fingertip applications due to peripheral arterial vasoconstriction under hypoxic conditions [28]. Since our device monitors blood oxygen saturation on the upper arm, hypoxic vasoconstriction should be less of a factor. The HeartRate3Click pulse oximetry module was selected for its inexpensive, prepackaged design, but the accuracy of heart rate measurements may be improved by using green light PPG in place of red-infrared to further minimize motion effects [25]. For our device, blood oxygen saturation was of interest for holistic health assessment, which cannot be achieved using green light PPG, and inclusion of green wavelengths was forgone to improve battery life. The penetration depth of red-infrared wavelengths is also favorable for a broader range of applications, in which arm girth and vascular depth could vary.

Our device is sensitive to changes in activity, galvanic skin response, and skin temperature. Monitoring trends in skin temperature provides information relevant to changes to core body temperature, making it a valuable tool for assessment of heat stress. The metrics of activity intensity and skin moisture, indicated by galvanic skin response, contribute to a more descriptive health overview. By considering these parameters together, relationships between an individual’s behavior and any resulting physiological effect can be assessed. The multi-parameter approach that our device provides gives it the flexibility for a multitude for personal and research applications, including determination of personal heat risk factors and when used in conjunction with other (e.g., ambient) monitoring data, documentation of changes that may occur in response to time-varying ambient conditions.

The compact design of this device improves the comfort and utility of health monitoring systems. When compared to other available systems, such as the Equivital LifeMonitor^TM^, the presented armband device offers the easy adaptability for individualized comfort. Though the Equivital offers accurate measurements of heart rate and heart rate variability via ECG, it is still susceptible to motion artifacts [29]. Proprietary software, Vivosense, can handle these artifacts, but at additional cost. Application of more common FFT techniques can be applied with our device data to extract meaningful information despite noise interference. Two-lead ECG, which is used by the Equivital, is also susceptible to considerations that are avoided by employing PPG, including electromyographic effects and crosstalk [29]. The chest harness design of the Equivital may be cumbersome for active individuals and difficult for elderly individuals to manipulate. Additional sensors can be attached to the Equivital device, but options such as a fingertip pulse oximeter require wired connection to the chest harness for assessment of blood oxygen saturation, which are unrealistic for real-world situations. The armband device provides enhanced comfort, convenience, and flexibility for a wide range of individuals and uses.

Low-cost options for personalized health monitoring are of particular interest to vulnerable populations, who may not have access to current commercial telehealth products. For elderly persons in institutions or living alone at home, such monitoring systems may be of particular benefit, and development of personal wearable technology for health monitoring can fill a critical health care need. Additionally, such technology can accelerate research into heat effects. With the emergence of health informatics and telehealth, low-cost, integrated systems that are comfortable and unobtrusive and that can be seamlessly integrated into daily life are needed [30]. Health risk, especially in the case of heat stress, is exacerbated by factors such as language barriers, migrant worker status, low income, and poor housing and healthcare [14]. A device, such as the one we propose, may offer solutions to mitigate health and heat illness risk.

Initial testing of this wearable demonstrates measurement capability of clinical parameters, such as skin temperature, heart rate, blood oxygen saturation, activity, and galvanic skin response. Future investigation will be needed to assess the performance of this device in individuals experiencing heat-related illnesses, exhibiting physiological extremes.

## 5. Conclusions

This proof-of-concept wearable multiparameter health monitoring device provides the opportunity for heat risk and physiological assessments in both research, clinical, and home settings. With convenient wear on the upper arm, the device offers a variety of versatile uses, especially with athletes, military personnel, and laborers, who engage in activities that may not be conducive to halter-type monitors, and for elderly individuals, who may have limitations in joint mobility that make other devices challenging to wear. Thus, for this latter population, the devices may be more practical for at-home monitoring and health assessment. Currently, the device requires manual data transfer from a microSD card, but when coupled with wireless technologies, such as WiFi or Bluetooth, this device presents the opportunity for direct-to-consumer, low-cost telehealth. Our platform is also adaptable to the addition of new sensors in the future, as well as integration with other available health monitoring devices. The capability of the already incorporated PPG sensor can also be extended to enable blood pressure and respiratory monitoring. Additional work can be done to survey users on the comfort of the device to improve and miniaturize future designs. New iterations of the device design may also replace commercial sensors with bespoke counterparts that utilize emerging biofabrication techniques to improve temperature and electrochemical sensing as well as miniaturization for comfort enhancements [31,32].

## Figures and Tables

**Table 1:** An intensity scale was defined according to a set of representative activities. The acceleration ranges for each activity were used to differentiate between activity levels.

Activity Level	Representative Activity	X-Axis Acceleration Range (mg)		Y-Axis Acceleration Range (mg)		Z-Axis Acceleration Range (mg)	
		Min	Max	Min	Max	Min	Max
1	Sitting at a desk	−7	1035	−89	1035	−312	−7
2	Walking at 2 mph	−7	1640	−480	753	−1093	160
3	Climbing stairs	−7	1925	−265	734	−511	363
4	Jogging at 6 mph	−656	1988	−1125	1988	−1390	722
5	Sprinting at 10 mph	−796	1988	−1980	1984	−1562	1906

**Table 2:** The confusion matrix for the SVM activity intensity classifier shows the classification accuracy for our methodology applied to data from multiple test subjects. Misclassification occurs solely between levels 2 and 3.

Predicted Class	1	4 25%	0 0.00%	0 0.00%	0 0.00%	0 0.00%	0 0.00%
	2	0 0.00%	1 6.25%	0 0.00%	0 0.00%	0 0.00%	0 0.00%
	3	0 0.00%	2 12.5%	3 18.75%	0 0.00%	0 0.00%	0 0.00%
	4	0 0.00%	0 0.00%	0 0.00%	3 18.75%	0 0.00%	0 0.00%
	5	0 0.00%	0 0.00%	0 0.00%	0 0.00%	3 18.75%	0 0.00%
		0 0.00%	0 0.00%	0 0.00%	0 0.00%	0 0.00%	87.5% 12.5%
		1	2	3	4	5	
		True Class					

## Supplementary Materials

The following are available online at https://www.mdpi.com/1424-8220/20/3/855/s1, Figure S1: Schematics for the main board of device, Figure S2: Schematics for the peripheral board of device.
