# Supplementary material for: Wearable Sensor System to Monitor Physical Activity and the Physiological Effects of Heat Exposure
Source: Sensors (Basel). 2020 Feb 6;20(3):855. doi: 10.3390/s20030855 (PMC7039288; doi:10.3390/s20030855)
Supplement: Supplementary file 1 [file sensors-20-00855-s001.pdf]

sensors

Supplementary

Wearable sensor system to monitor physical activity and the physiological effects of heat exposure

Sean Pham [1], Danny Yeap [1], Gisela Escalera [2], Rupa Basu [3], Xiangmei Wu [3], Nicholas J. Kenyon [4,5,6], Irva Hertz-Picciotto [7], Michelle J. Ko [8] and Cristina E. Davis [1,*]

1 Department of Mechanical and Aerospace Engineering, University of California, Davis, CA 95616, USA.; sapham@ucdavis.edu (S.P.); dyeap@ucdavis.edu (D.Y.)
2 Center for Healthcare Policy and Research, University of California, Davis, CA 95616, USA.; gescalera@ucdavis.edu (G.E.)
3 California Environmental Protection Agency, California Office of Environmental Health Hazard Assessment, 1515 Clay Street, Oakland, CA 94612, USA.; Rupa.Basu@oehha.ca.gov (R.B.); Xiangmei.Wu@oehha.ca.gov (X.W.)
4 Department of Internal Medicine, 4150 V Street, Suite 3400, University of California, Davis, Sacramento, CA 95817, USA.; njkenyon@ucdavis.edu
5 Center for Comparative Respiratory Biology and Medicine, University of California, Davis, CA 95616, USA.
6 VA Northern California Health Care System, 10535 Hospital Way, Mather, CA 95655, USA.
7 Division of Environmental and Occupational Health, Department of Public Health, University of California, Davis, CA 95616, USA.; iher@ucdavis.edu
8 Center for Regional Change, Department of Public Health, University of California, Davis, CA 95616, USA.; mijko@ucdavis.edu
* Correspondence: cedavis@ucdavis.edu

Received: 16 December 2019; Accepted: 30 January 2020; Published: date

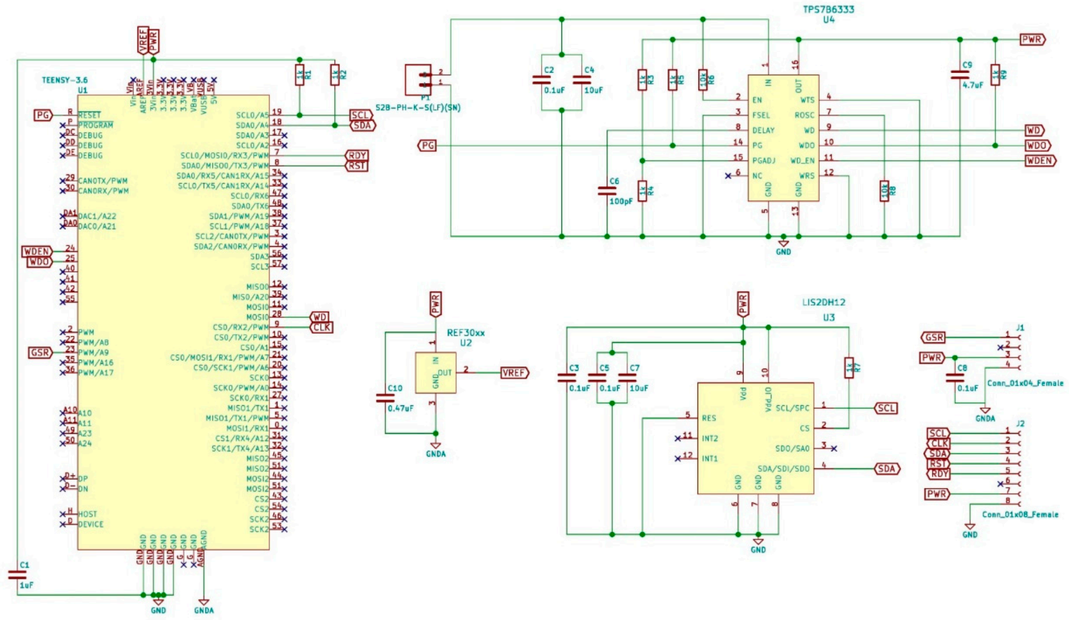

Figure S1. Schematics for the main board of device, which contains the power and control elements of the device.

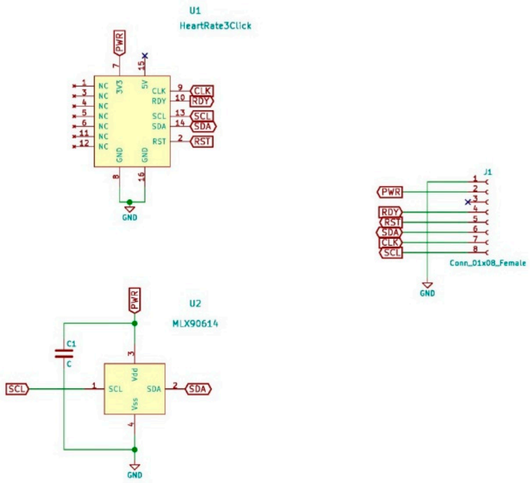

Figure S2. Schematics for the peripheral board of device, which contains the optical pulse oximetry and temperature sensors.
